# Combined TLR2/4-Activated Dendritic/Tumor Cell Fusions Induce Augmented Cytotoxic T Lymphocytes

**DOI:** 10.1371/journal.pone.0059280

**Published:** 2013-03-15

**Authors:** Shigeo Koido, Sadamu Homma, Masato Okamoto, Yoshihisa Namiki, Kazuki Takakura, Akitaka Takahara, Shunichi Odahara, Shintaro Tsukinaga, Toyokazu Yukawa, Jimi Mitobe, Hiroshi Matsudaira, Keisuke Nagatsuma, Kan Uchiyama, Mikio Kajihara, Seiji Arihiro, Hiroo Imazu, Hiroshi Arakawa, Shin Kan, Hideo Komita, Masaki Ito, Toshifumi Ohkusa, Jianlin Gong, Hisao Tajiri

**Affiliations:** 1 Division of Gastroenterology and Hepatology, Department of Internal Medicine, The Jikei University School of Medicine, Tokyo, Japan; 2 Institute of Clinical Medicine and Research, The Jikei University School of Medicine, Tokyo, Japan; 3 Department of Oncology, Institute of DNA Medicine, The Jikei University School of Medicine, Tokyo, Japan; 4 Division of Cellular Signaling, Institute for Advanced Medical Research, Keio University School of Medicine, Tokyo, Japan; 5 Department of Endoscopy, The Jikei University School of Medicine, Tokyo, Japan; 6 Department of Medicine, Boston University School of Medicine, Boston, Massachusetts, United States of America; Leiden University Medical Center, Netherlands

## Abstract

Induction of antitumor immunity by dendritic cell (DC)-tumor fusion cells (DC/tumor) can be modulated by their activation status. In this study, to address optimal status of DC/tumor to induce efficient antigen-specific cytotoxic T lymphocytes (CTLs), we have created various types of DC/tumor: 1) un-activated DC/tumor; 2) penicillin-killed *Streptococcus pyogenes* (OK-432; TLR4 agonist)-activated DC/tumor; 3) protein-bound polysaccharides isolated from *Coriolus versicolor* (PSK; TLR2 agonist)-activated DC/tumor; and 4) Combined OK-432- and PSK-activated DC/tumor. Moreover, we assessed the effects of TGF-β1 derived from DC/tumor on the induction of MUC1-specific CTLs. Combined TLR2- and TLR4-activated DC/tumor overcame immune-suppressive effect of TGF-β1 in comparison to those single activated or un-activated DC/tumor as demonstrated by: 1) up-regulation of MHC class II and CD86 expression on DC/tumor; 2) increased fusion efficiency; 3) increased production of fusions derived IL-12p70; 4) activation of CD4^+^ and CD8^+^ T cells that produce high levels of IFN-γ; 5) augmented induction of CTL activity specific for MUC1; and 6) superior efficacy in inhibiting CD4^+^CD25^+^Foxp3^+^ T cell generation. However, DC/tumor-derived TGF-β1 reduced the efficacy of DC/tumor vaccine *in vitro*. Incorporating combined TLRs-activation and TGF-β1-blockade of DC/tumor may enhance the effectiveness of DC/tumor-based cancer vaccines and have the potential applicability to the field of adoptive immunotherapy.

## Introduction

Dendritic cells (DCs) are specialized antigen-presenting cells (APCs) and attractive vectors for cancer immunotherapy [1]. DC-tumor fusion cells (DC/tumor) are able to directly present a broad spectrum of tumor-associated antigens (TAAs), including those known and unidentified, through major histocompatibility complex (MHC) class I and class II molecules in the context of costimulatory molecules [2]. Both un-activated and activated DC/tumor are capable of inducing cytotoxic T lymphocytes (CTLs), however, little is known which activation state of DC/tumor is powerful tool for cancer vaccines.

Toll-like receptors (TLRs) have recently emerged as key receptors responsible for recognizing specific conserved components of microbes and can trigger DC activation and cytokine production, thus effectively bridging innate and adaptive immunity [3], [4]. Incubation with a single TLR agonist induces the expression of approximately 1% of global gene transcripts, however, gene expression is increased more than 5-fold after synergistic TLR-stimulation [5]. Thus, full activation of DC/tumor may require the assembly of receptor signaling complexes by combined TLR agonists. A protein-bound polysaccharide isolated from *Coriolus versicolor* (PSK) acts as a TLR2 agonist and can activate DCs, T cells, and natural killer (NK) cells [6], [7]. Penicillin-killed and lyophilized preparations of a low-virulence strain (Su) of *Streptococcus pyogenes* (OK-432) act as a TLR4 agonist and can activate DCs, macrophages, neutrophils, T cells, and NK cells by inducing multiple cytokines such as interleukin (IL)-12 and interferon (IFN)-γ and polarizing T cell responses to a Th1-dominant state [8]. Both OK-432 and PSK are good manufacturing practice (GMP) grade agents and have been used clinically as biological response modifiers [9], [10]. To assess which activation state of DC/tumor is powerful tool for induction of efficient CTL responses, we used combined OK-432 and PSK. Moreover, effects of DC/tumor derived immune-suppressive factors such as transforming growth factor (TGF)-β1 on CTL induction were also assessed.

We show that DC/tumor activated with combined TLR2 and TLR4 are most effective inducer of MUC1-specific CTL activation compared with solitary TLR2- or TLR4-activated DC/tumor on a per fusion cell basis. However, DC/tumor-derived TGF-β1 reduces the efficacy of CTL induction, even when stimulated with combined TLR2 and TLR4 agonists *in vitro*.

## Materials and Methods

### Tumor cells and conditioned medium

The tumor cell lines, PANC-1 (HLA-A2^+^MUC1^+^), MIA PaCa-2 (HLA-A2^-^MUC1^+^), and K562 (HLA-A2^-^MUC1^+^) were purchased from American Type Culture Collection (ATCC, Manassas, VA) and maintained in DMEM supplemented with 100 U/mL penicillin, 100 µg/mL streptomycin and 10% fetal calf serum (FCS). To establish a stable transfectant of PANC-1 cells expressing high levels of TGF-β1, the human TGF-β1 coding region was cloned from pCMV-SPORT6 (Open Biosystems, Lafayette, CO) and the fragment was inserted to a *Nhe*I*/Hin*dIII site of pcDNA3.1(+). The constructed plasmid was transfected to PANC-1 cells using Lipofectamine 2000 transfection reagent (Life Technologies, Tokyo, Japan). Transfected clones were selected with 1 mg/mL geneticin (G418; Life Technologies). TGF-β1 mRNA levels were determined by real-time PCR and the protein production was confirmed by ELISA (R&D Systems, Minneapolis, MN) (Fig. S1). A clone exhibiting the highest production of TGF-β1 protein (PANC/TGF-β) was selected for further analyses. Next, to avoid the potential hazards of FCS that limit safety in clinical trials, the FCS-independent tumor cells were established and cultured in TIL Media I (IBL, Gunma-ken, Japan) supplemented with 10% human plasma protein fraction (PPF) (Baxter Healthcare Corp., Tokyo, Japan) [11]. PANC-1 cells transfected with an empty vector (PANC/Mock) and un-transfected PANC-1 cells were used as controls.

### Generation of monocyte-derived DCs

Peripheral blood mononuclear cells (PBMCs) from healthy donors (HLA-A*0201^+^) were obtained with individual written informed consent. The study protocol was reviewed and approved by the ethics committee of the Jikei Institutional Review Board, Jikei University School of Medicine, as well as the clinical study committee of the Jikei University Kashiwa hospital (No. 14-60 (3209)). Briefly, PBMCs were isolated from peripheral blood by Ficoll density-gradient centrifugation and incubated in tissue culture flasks at 37°C for 30 min in AIM V (Life Technologies Japan Ltd.) supplemented with 1% heat-inactivated autologous serum. Adherent PBMCs were cultured for 4 days in AIM V supplemented with 1000 U/mL GM-CSF (PeproTech, Rocky Hill, NJ) and 500 U/mL IL-4 (Diaclone Research, Boulevard Fleming, France) to generate immature DCs (Imm-DCs). On day 4, Imm-DCs were activated with 0.1 KE/mL (0.1 KE equals 10 µg of dried *streptococci*) OK-432 (Chugai Pharmaceutical, Tokyo, Japan) (OK-DCs), 100 µg/mL PSK (Kureha Corp., Tokyo, Japan) (PSK-DCs) or both (OPK-DCs) for additional 2 days.

### Fusion of DCs and tumor cells

We developed various types of DC/tumor by alternating fusion partners as follows: Imm-DCs fused with PANC/Mock (Imm/Mock); OK-DCs fused with PANC/Mock (OK/Mock); PSK-DCs fused with PANC/Mock (PSK/Mock); OPK-DCs fused with PANC/Mock (OPK/Mock); Imm-DCs fused with PANC/TGF-β (Imm/TGF-β); OK-DCs fused with PANC1/TGF-β (OK/TGF-β); PSK-DCs fused with PANC/TGF-β (PSK/TGF-β); and OPK-DCs fused with PANC/TGF-β (OPK/TGF-β). Briefly, tumor cells and DCs were mixed at a ratio of 1∶10 and fusions were generated using 50% polyethylene glycol (PEG) (Sigma-Aldrich, St Louis, MO) [12]. Fusions generated with Imm-DCs were maintained in fusion medium (AIM V supplemented with 1000 U/mL GM-CSF, 500 U/mL IL-4, and 10% PPF). Moreover, fusions generated with OK-DCs, PSK-DCs, or OPK-DCs were activated with fusion medium in the presence of 0.1 KE/mL OK-432, 100 µg/mL PSK, or both, respectively. After 2 days culture, each DC/tumor were stimulated, integrated to a single entity, and purified by gentle pipetting [12].

### Phenotype analysis

Cells were incubated with FITC-conjugated monoclonal antibodies (mAbs) against MUC1 (HMPV; BD Pharmingen, San Jose, CA), MHC class I (W6/32), MHC class II (HLA-DR), B7-1 (CD80), B7-2 (CD86), TLR2 (CD282), TLR4 (CD284) (BD Pharmingen), HLA-A2 (One Lambda, Canoga Park, CA), or matched isotype control IgG. DC populations were gated based on their forward- *vs.* side-scatter profile then analyzed for expression of MHC class I, MHC class II, CD80, CD86, CD83, CCR7, and MUC1. For dual expression in DC/tumor, incubation was performed with FITC-conjugated mAbs against MUC1 and PE-conjugated mAbs against HLA-DR or CD86. The cell aggregations were eliminated by gating out before FACS analysis [12]. The DC/tumor were then determined by FACS analysis, where the fused cells were identified as MUC1^+^HLA-DR^+^ or MUC1^+^CD86^+^. To assess the forkhead box P3 (Foxp3) expression in the CD4^+^CD25^high^ T cells, stimulated T cells were incubated with FITC-conjugated mAbs against CD25 (2A3; BD Pharmingen) and PE-Cy-5-conjugated mAbs against CD4 (RPA-T4; BD Pharmingen). After washing, cells were fixed for 10min and permeabilized for 30min using the human Foxp3 Buffer Set (BD Pharmingen), then stained with PE-conjugated mAbs against Foxp3 (259D/C7; BD Pharmingen) or matched isotype control IgG. To identify Foxp3^+^ cells in the CD4^+^CD25^high^ T cells, T cell populations were gated based on their forward- *vs.* side-scatter profile. CD4^+^CD25^high^ T cells were then analyzed for Foxp3 expression. Cells were fixed with 2% paraformaldehyde, and analyzed by BD FACSCalibur flow cytometer (Beckton Dickinson, Mountain View, CA) using FlowJo analysis software (Tree Star, OR, USA).

### T cell stimulation

The numbers of DC/tumor were described based on the number of cells that coexpressed HLA-DR and MUC1 in the fusion cell preparations. Equal numbers of DC/tumor (HLA-A2^+^) were cocultured with autologous nonadherent PBMCs (HLA-A2^+^) at a ratio of 1∶10 in the absence of recombinant human (rh)IL-2 for 3 days and purified through nylon wool to remove APCs. From day 4, a low dose of rhIL-2 (10 U/mL; Shionogi, Osaka, Japan) was added and maintained until day 7. DCs alone, tumor cells alone, and DCs mixed with tumor cells were used as controls.

### Enzyme-linked immunosorbent assay (ELISA)

DC/tumor (1×10^5^ cells/mL/well), DCs (1×10^5^ cells/mL/well), or tumor cells (1×10^4^ cells/mL/well) were cultured for 48hr. Supernatants from these cells were tested for IL-12p70, the active form of TGF-β1 (R&D Systems), or heat shock protein (HSP)90α (**Enzo Life Sciences,** Farmingdale, NY**)** by ELISA. The minimum detectable dose of human IL-12p70 is typically less than 0.5 pg/mL. To measure the total (latent and active) amount of TGF-β1, the latent form was converted to the active form by treatment with hydrochloric acid. The active form of TGF-β1 was analyzed directly by ELISA.

### Analysis of cytokine producing CD4^+^ and CD8^+^ T cells

Stimulated T cells were harvested by nylon wool separation for analysis of human IFN-γ or IL-10 production using each cytokine secretion assay kit according to manufacturer’s instructions (Miltenyi Biotec, Auburn, CA). Briefly, T cells were incubated with IFN-γ or IL-10 catching reagent for 5min at 4°C and cultured in RPMI supplemented with 10% PPF for 45min. Cells were then stained with PE-conjugated anti-IFN-γ or IL-10 detection mAbs and FITC-conjugated mAbs against CD4 or CD8 (BD Pharmingen), washed, fixed with 2% paraformaldehyde, and analyzed by flow cytometry using FlowJo analysis software. T cell populations were gated based on their forward- *vs.* side-scatter profile. CD4^+^ or CD8^+^ T cell populations were gated then the percentage of IFN-γ-positive CD4^+^ or CD8^+^ T cells among the whole CD4^+^ or CD8^+^ T cells was assessed.

### Pentamer staining

Stimulated T cells were harvested by nylon wool separation then incubated with PE-conjugated MUC1 pentamer (HLA-A*0201, STAPPVHNV) (Proimmune, Oxford, UK) for 1hr at 4°C. After washing, the T cells were stained with FITC-conjugated mAbs against CD8 (BD Pharmingen), washed, fixed with 2% paraformaldehyde, and analyzed by flow cytometry using FlowJo analysis software. Complexes of PE-irrelevant pentamers were used as controls. T cell populations were gated based on their forward- *vs.* side-scatter profile. CD8^+^ T cell populations were gated then percentage of MUC1 pentamer-positive CD8^+^ T cells among the whole CD8^+^ T cells was assessed.

### Cytotoxicity assays

The cytotoxicity assays were performed by flow cytometric analysis using Active Caspase-3 Apoptosis kit I (BD Pharmingen) that measured CTL-induced caspase-3 activation in target cells by detecting the specific cleavage of fluorogenic caspase-3 [13]. Briefly, target cells were labeled with PKH-26 (Sigma-Aldrich), washed, cultured with stimulated T cells for 2h at 37°C in 96-well, V-bottomed plates at the indicated effector cell:T cell (E:T) ratios. Cells were fixed with Cytofix/Cytoperm Solution (BD Pharmingen), washed with Perm/Wash Buffer (BD Pharmingen), and incubated with FITC-conjugated mAbs against human active caspase-3 substrate (BD Pharmingen) for 30 min at room temperature, followed by two washes with Perm/Wash buffer. In certain experiments, PANC-1 cells were preincubated with anti-HLA-A2 (One Lambda) or control IgG for 30min at 37°C before the addition of effector cells. The percentage of cytotoxicity (mean ± SD of three replications) was determined with the following equation: percentage of caspase-3 staining  =  (caspase-3^+^PKH-26^+^ cells)/(caspase-3^+^PKH-26^+^ cells + caspase-3^-^PKH-26^+^ cells) × 100.

### Statistical analysis

Results are expressed as mean ± SD as indicated in the legends. We used one-way analysis of variance to determine significance. When P-values were 0.05 or less, differences were considered statistically significant.

## Results

### Characterization of DCs activated with combined OK-432 and PSK

**Figure 1:**
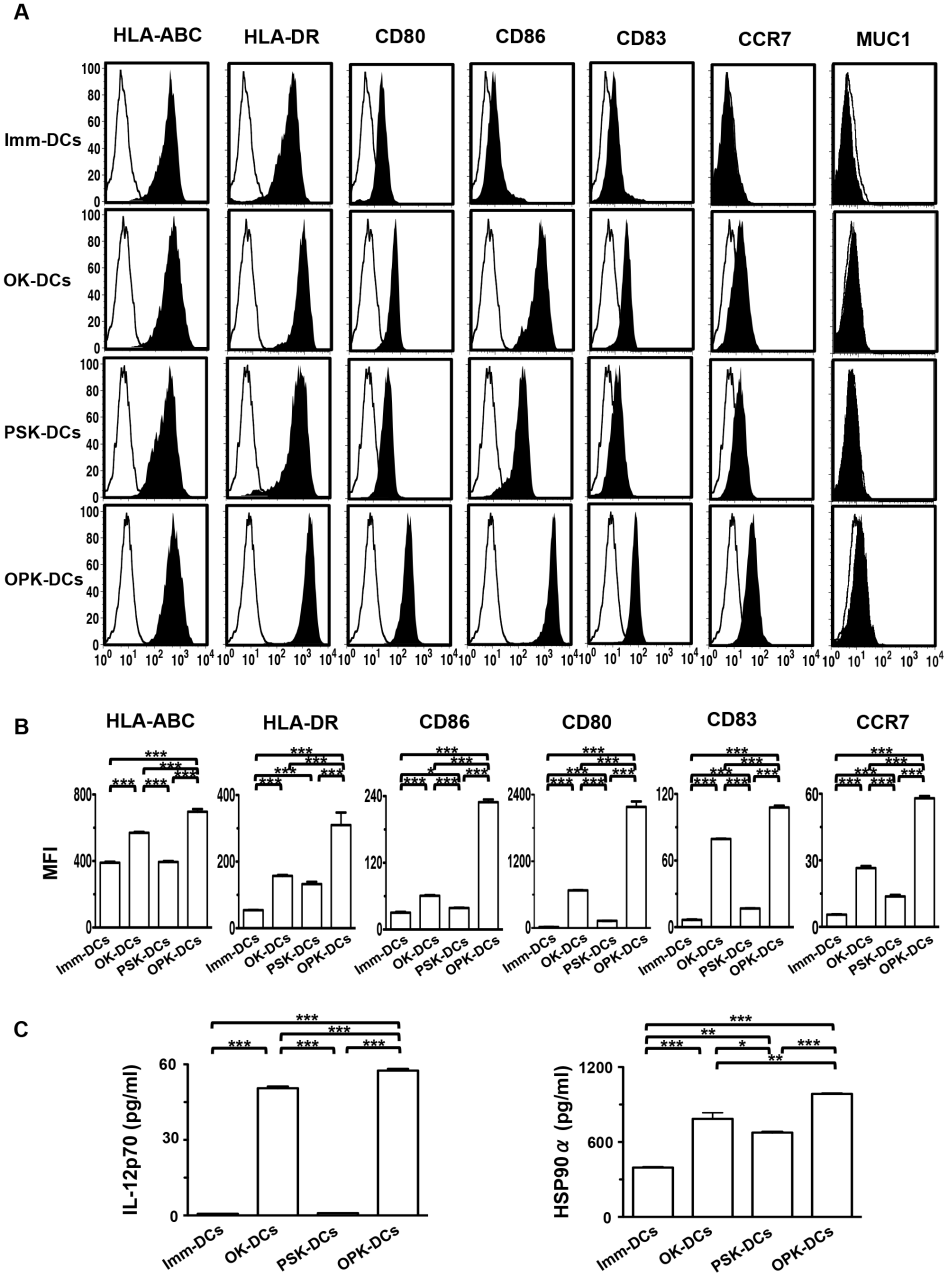
Phenotypic and functional characterization of DCs. (A) Imm-DCs, OK-DCs, PSK-DCs, and OPK-DCs were analyzed by flow cytometry for expression of the indicated antigens (n = 5). Unfilled histogram profile indicates the isotype control, and solid histogram indicates the specific antibody. (B) MFI of the indicated molecules expressed by DCs (Imm-DCs, OK-DCs, PSK-DCs, and OPK-DCs) was analyzed (n = 5). (C) Production of IL-12p70 or HSP90α by DCs (Imm-DCs, OK-DCs, PSK-DCs, and OPK-DCs at 1×10^5^/mL) (n = 5) was analyzed by ELISA. The results are expressed as the mean ± SD. ***P<0.001; **P<0.01; *P<0.05.

Imm-DCs displayed a characteristic phenotype with the expression of HLA-ABC, HLA-DR, CD80, and CD86, but low levels of CD83, and little or none of CCR7 and MUC1 (Fig. 1A). OK-DCs and PSK-DCs up-regulated the expression of HLA-DR, CD80, CD86, and CCR7, compared with Imm-DCs, although OPK-DCs expressed the most active phenotype (Fig. 1A and B). Moreover, OK-DCs exhibited increased production of IL-12p70, compared with Imm-DCs or PSK-DCs (Fig. 1C). Interestingly, OPK-DCs showed enhanced production of IL-12p70 and extracellular HSP90α, compared to Imm-DCs, OK-DCs, or PSK-DCs (Fig. 1C). In addition, there was no TGF-β1 production by either type of DC (data not shown). These results suggest that OPK-DCs are the most activated, compared with Imm-DCs, OK-DCs, or PSK-DCs.

### Establishment of a stable transfectant of PANC-1 cells expressing TGF-β1

To examine the HLA-A2 restrictive MUC1-specific CTLs induction by DC/tumor, we used the MUC1 and HLA-A2 positive tumor cell line, PANC-1 cells. Little or none of the active form of TGF-β1, but not IL-12p70 were detected in culture supernatants of PANC-1 cells (data not shown). To clearly assess the effects of TGF-β1 on CTL induction by DC/tumor, we established a stable transfectant of PANC-1 cells expressing high levels of TGF-β1 (PANC/TGF-β1#1) (Fig. S1). The TGF-β1 mRNA expression level in PANC/TGF-β1#1 was much higher than in the cell line transfected with the expression vector alone (PANC/Mock) as well as PANC-1 cells (data not shown). The total (latent and active) levels of TGF-β1 in culture supernatants from the tumor cells inhibited Mv1Lu cell growth (Fig. S2A). While the active form of TGF-β1 in the supernatant from PANC/TGF-β1#1 inhibited Mv1Lu cell growth, the supernatants from PANC/Mock did not (Fig. S2B). There was no difference in inhibition of Mv1Lu cell growth using acid-treated or un-acid-treated supernatants from PANC/Mock or PANC-1 cells (data not shown). Therefore, we used PANC/TGF-β1#1 as a stable transfectant of PANC-1 cells expressing high levels of TGF-β1 (PANC/TGF-β) in the following experiments.

### Characterization of tumor cells stimulated with combined OK-432 and PSK

**Figure 2:**
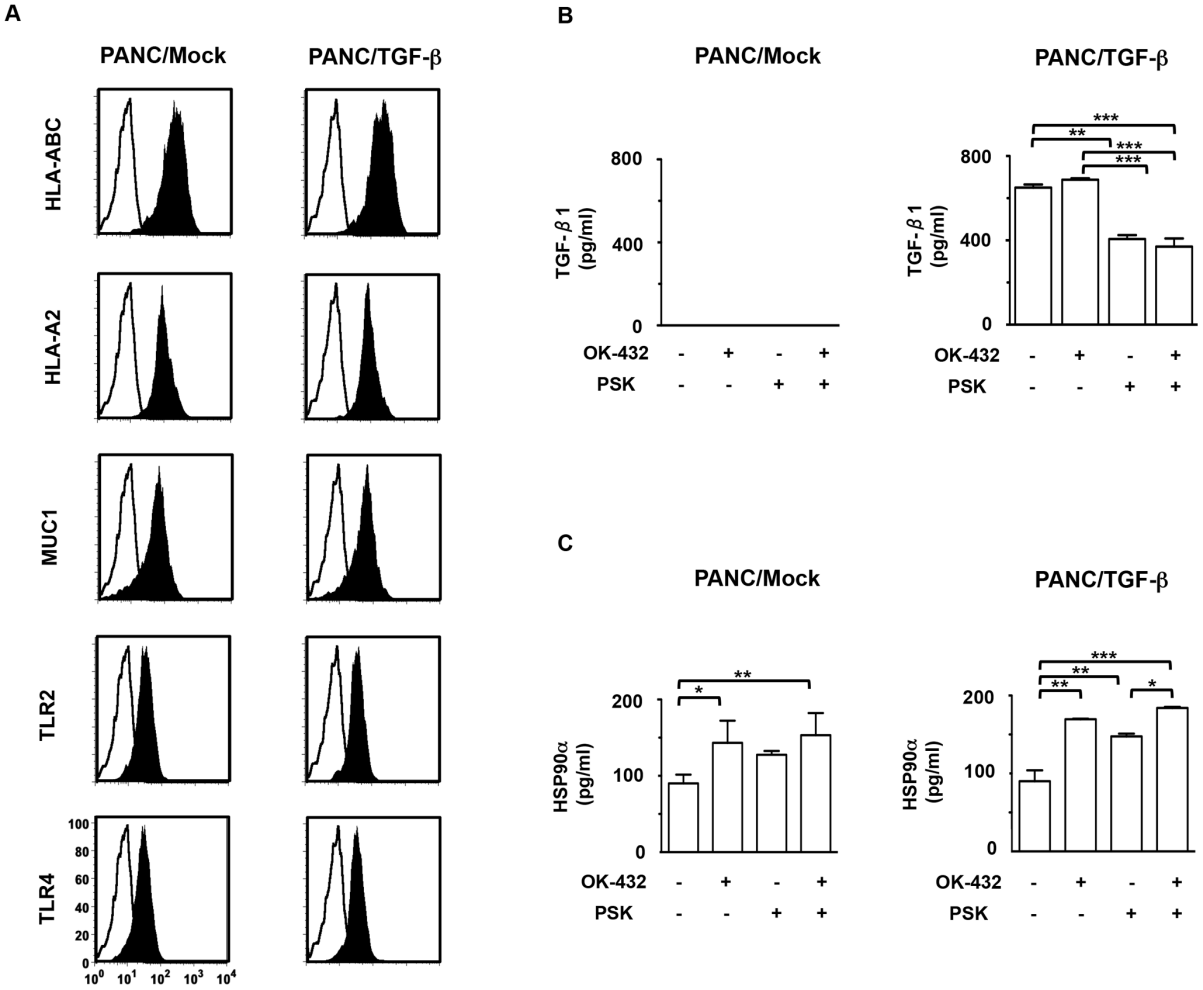
Phenotypic and functional characterization of tumor cells. (A) PANC/Mock and PANC/TGF-β were analyzed by flow cytometry for expression of the indicated antigens. Unfilled histogram profile indicates the isotype control, and solid histogram indicates the specific antibody. PANC/Mock or PANC/TGF-β1 (1×10^4^/mL) were cultured with OK-432 or PSK or both for 48hr. Mean concentration of (B) the active form of TGF-β1 or (C) HSP90α was analyzed by ELISA. The results are expressed as the mean ± SD. ***P<0.001; **P<0.01; *P<0.05.

HLA-ABC, HLA-A2, MUC1, TLR2, and TLR4 were expressed at the same level in PANC/TGF-β, PANC/Mock (Fig. 2A), and PANC-1 cells (data not shown). Little or none of the active form of TGF-β1 was detected in culture supernatants of PANC/Mock (Fig. 2B, left panel). Although production of high levels of the active form of TGF-β1 was detected in PANC/TGF-β as well as OK-432-stimulated PANC/TGF-β, the production was significantly decreased by stimulation with PSK (Fig. 2B, right panel). Interestingly, increased levels of HSP90α production were detected in PANC/TGF-β stimulated with OK-432, PSK, or both, compared to un-stimulated tumor cells (Fig. 2C). These findings suggest that the decreased TGF-β1 production in PSK-treated PANC/TGF-β1 may up-regulate its immunogenicity.

### Characterization of DC/tumor

**Figure 3:**
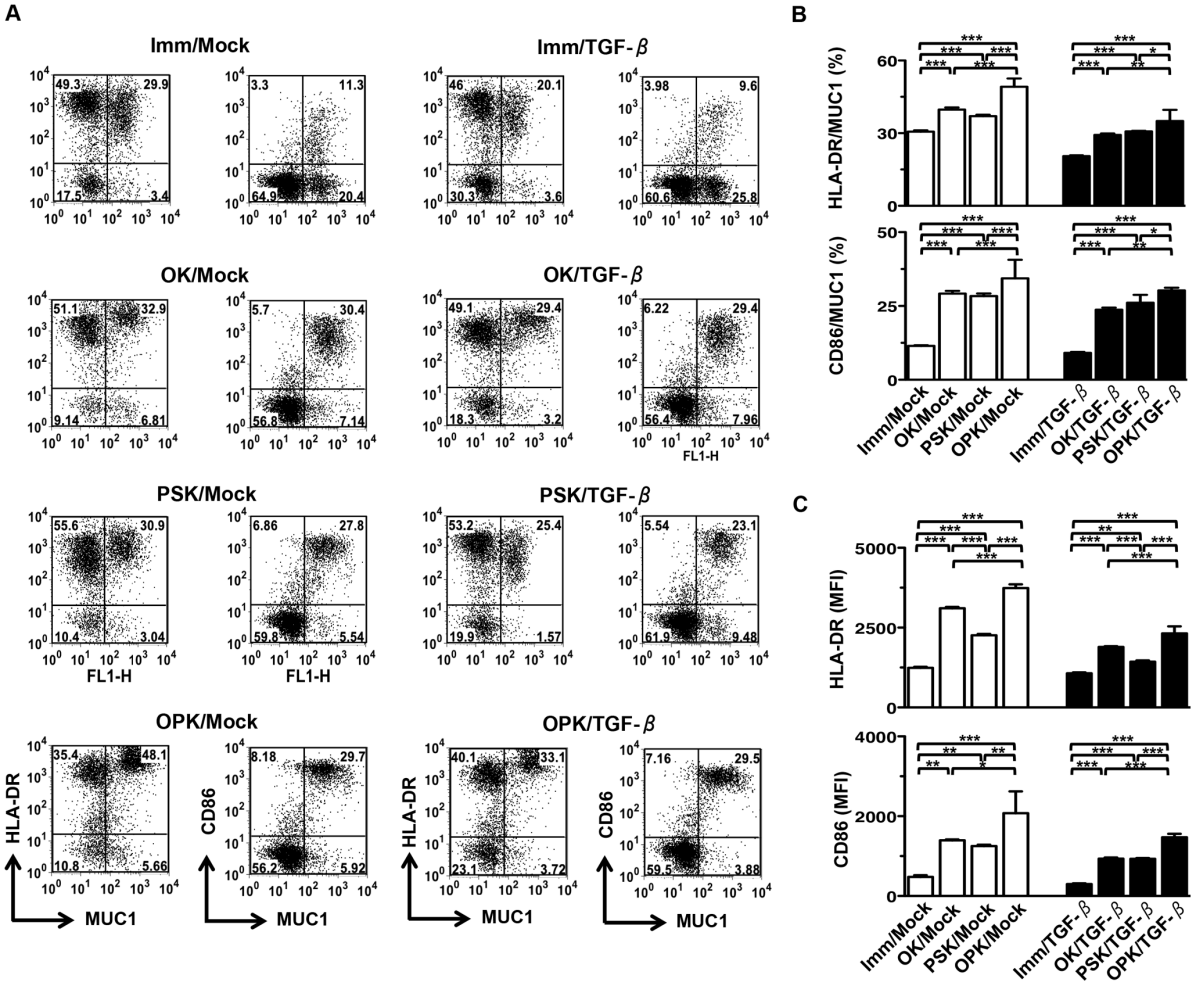
Phenotypic characterization of DC/tumor. (A) Eight types of DC/tumor (Imm/Mock, OK/Mock, PSK/Mock, OPK/Mock, Imm/TGF-β, OK/TGF-β, PSK/TGF-β, and OPK/TGF-β) (n = 4) were stained with FITC-conjugated mAbs against MUC1 and PE-conjugated mAbs against HLA-DR or CD86 and analyzed by two-color flow cytometry. The numbers of events in each region are shown. (B) Percentage of cells positive for both MUC1 and HLA-DR or CD86 in eight types of DC/tumor (Imm/Mock, OK/Mock, PSK/Mock, OPK/Mock, Imm/TGF-β, OK/TGF-β, PSK/TGF-β, and OPK/TGF-β) (n = 4) were analyzed. (C) Eight types of DC/tumor (Imm/Mock, OK/Mock, PSK/Mock, OPK/Mock, Imm/TGF-β, OK/TGF-β, PSK/TGF-β, and OPK/TGF-β) (n = 4) were stained with FITC-conjugated mAbs against MUC1 and PE-conjugated mAbs against HLA-DR or CD86 and analyzed by FACS, where the fused cells were identified as MUC1^+^HLA-DR^+^ or MUC1^+^CD86^+^. MFI of MUC1^+^HLA-DR^+^ or MUC1^+^CD86^+^ populations was analyzed. The results are expressed as the mean ± SD. ***P<0.001; **P<0.01; *P<0.05.

To examine the effects of the active form of TGF-β1 produced by DC/tumor on CTL induction, we developed eight types of DC/tumor. Tumor cells were successfully fused with different types of DCs (Fig. 3A). The fusion efficiency was determined by the percentage of MUC1 and HLA-DR or CD86 double-stained cells (Fig. 3B). OPK-DCs enhanced fusion efficiency compared to OK-DCs or PSK-DCs fusions generated with PANC/Mock as well as PANC/TGF-β (Fig. 3B). However, OPK/TGF-β showed decreased fusion efficiency, as compared to OPK/Mock (Fig. 3B).

Next, to assess the phenotypic characterization of DC/tumor, the mean fluorescence intensity (MFI) of HLA-DR and CD86 expression by DC/tumor were determined by FACS analysis, where the fused cells were identified as MUC1^+^HLA-DR^+^ or MUC1^+^CD86^+^. Although OK/Mock and PSK/Mock displayed higher MFI levels of HLA-DR and CD86 than Imm/Mock, OPK/Mock had the most active phenotype on a per fusion cell basis (Fig. 3C). Fusions generated with PANC/TGF-β and DCs exhibited lower MFI levels of HLA-DR and CD86 compared with PANC/Mock (Fig. 3C).

**Figure 4:**
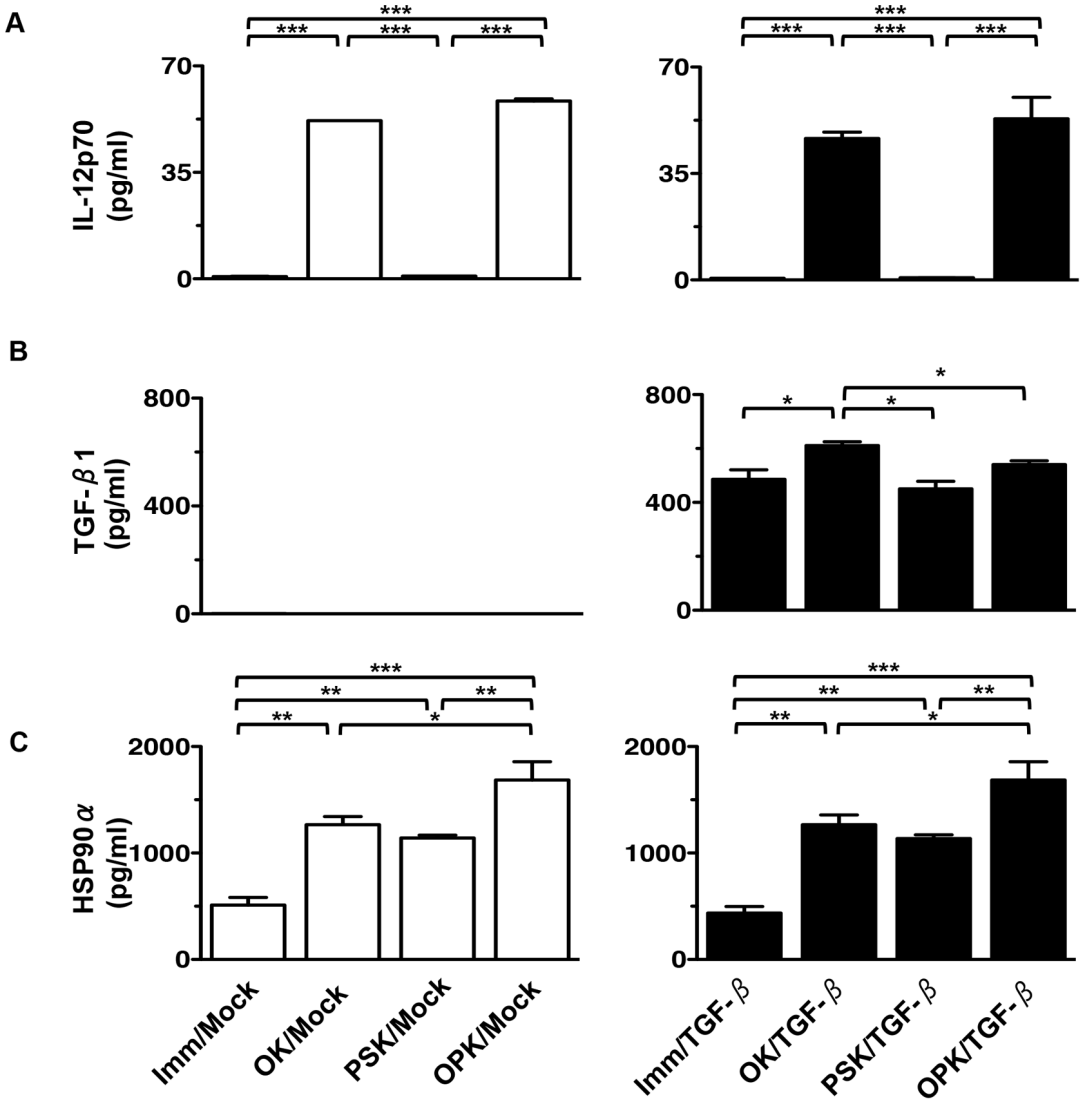
Functional characterization of DC/tumor. The mean concentrations of (A) IL-12p70, (B) the active form of TGF-β1, and (C) HSP90α derived from eight types of DC/tumor (Imm/Mock, OK/Mock, PSK/Mock, OPK/Mock, Imm/TGF-β, OK/TGF-β, PSK/TGF-β, and OPK/TGF-β) (n = 4) were analyzed by ELISA. The results are expressed as the mean ± SD. ***P<0.001; **P<0.01; *P<0.05.

Furthermore, we assessed the production of IL-12p70, the active form of TGF-β1, and HSP90α in supernatants from DC/tumor. High levels of IL-12p70 production were observed in OK/Mock and OPK/Mock, compared with Imm/Mock or PSK/Mock. IL-12p70 production in OPK/TGF-β was decreased compared to OPK/Mock (Fig. 4A), suggesting the active form of TGF-β1 suppressed the function of DCs in the fused cell complex, and thus inhibiting IL-12p70 production. As expected, the active form of TGF-β1 was produced at higher levels in fusions of PANC/TGF-β and all types of DCs, compared with PANC/Mock. Although the active form of TGF-β1 was significantly increased by OK/TGF-β, compared to Imm/TGF-β, the production was decreased by OPK/TGF-β (Fig. 4B). Interestingly, fusions generated with OPK-DCs produced increased levels of HSP90α, compared to those generated with other types of DCs (Fig. 4C). In addition, there was no difference in the production of IL-12p70, the active form of TGF-β1, and HSP90α between fusions generated with PANC/Mock or PANC-1 cells (data not shown). Collectively, these results suggest that up-regulated production of IL-12p70 and HSP90α in combined OK-432- and PSK-activated DC/tumor may increase their immunogenicity.

### T cell activation by DC/tumor

**Figure 5:**
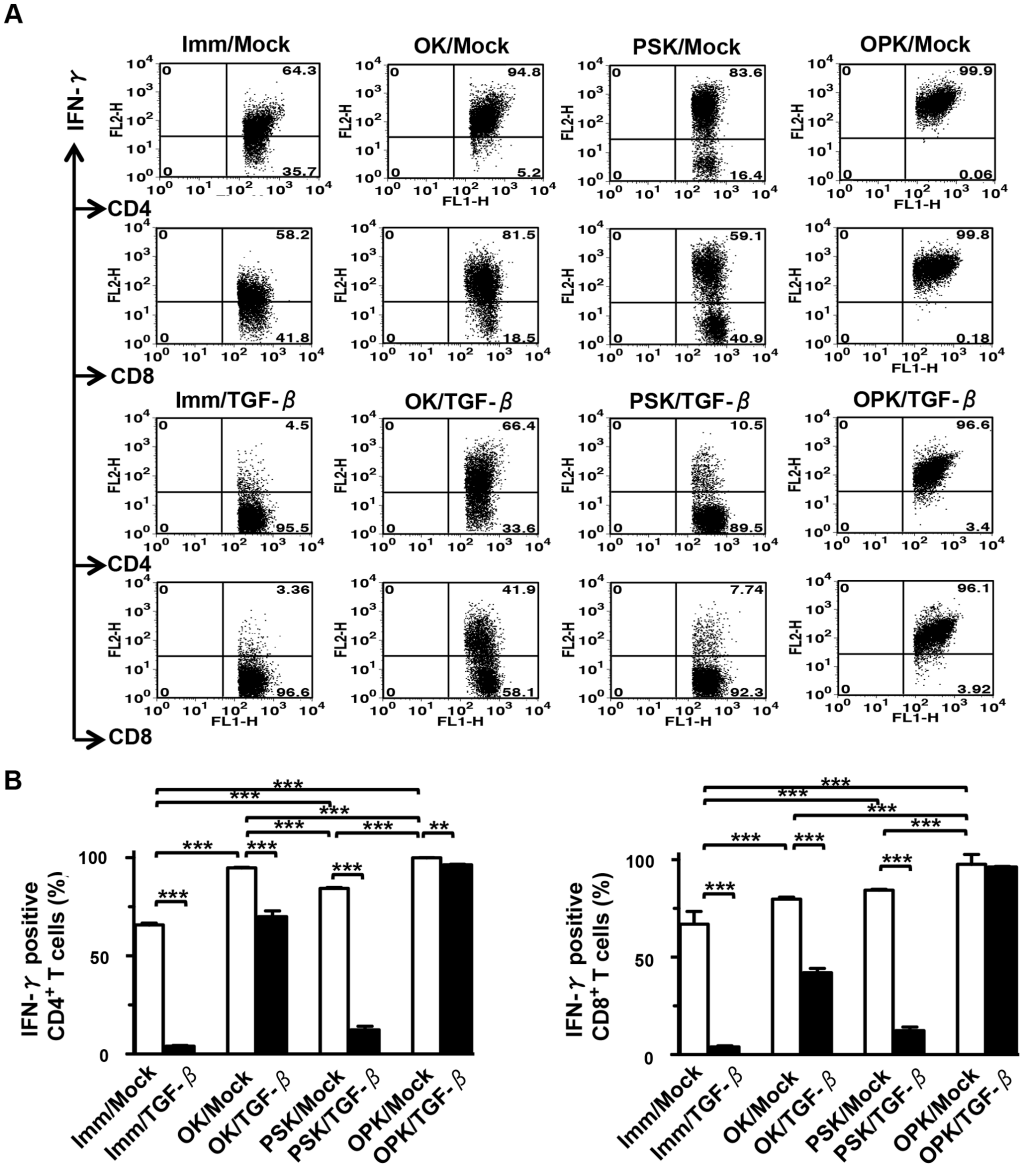
T cell stimulation by DC/tumor. (A) T cells (n = 5) were stimulated by eight types of DC/tumor in the absence of rhIL-2 for 3 days and then maintained in the presence of low doses of rhIL-2 for 7 days and assessed for IFN-γ production by flow cytometry. The numbers of events in gated CD4^+^ or CD8^+^ T cell populations are shown. (B) The percentage of IFN-γ-positive T cells among the gated CD4^+^ or CD8^+^ T cells was assessed (n = 5). The results are expressed as the mean ± SD. ***P<0.001; **P<0.01; *P<0.05.

OK/Mock and PSK/Mock induced higher levels of IFN-γ in CD4^+^ and CD8^+^ T cells compared to Imm/Mock. However, both OK/TGF-β1 and PSK/TGF-β1 reduced the numbers of IFN-γ-producing CD4^+^ and CD8^+^ T cells (Fig. 5A and B). Importantly, combined OK-432- and PSK-activated DC/tumor vigorously induced IFN-γ-producing CD4^+^ and CD8^+^ T cells (Fig. 5A and B). In contrast, there was little, if any, IFN-γ production by T cells cocultured with OPK-DCs mixed with tumor cells (data not shown). The low levels of IL-10 production by CD4^+^ and CD8^+^ T cells stimulated by DC/tumor did not impair the production of IFN-γ (data not shown). Although fusions stimulated with solitary OK-432 or PSK could stimulate both CD4^+^ and CD8^+^ T cells, by themselves they could not induce full stimulation of T cells. Taken together, our results show that combined OK-432- and PSK-activation of DC/tumor contributes to enhance their ability through a fusion process to stimulate IFN-γ-producing CD4^+^ and CD8^+^ T cells and may enable stronger CTL responses to be generated.

### Expression of Foxp3 in CD4^+^CD25high^+^T cells stimulated by DC/tumor

**Figure 6:**
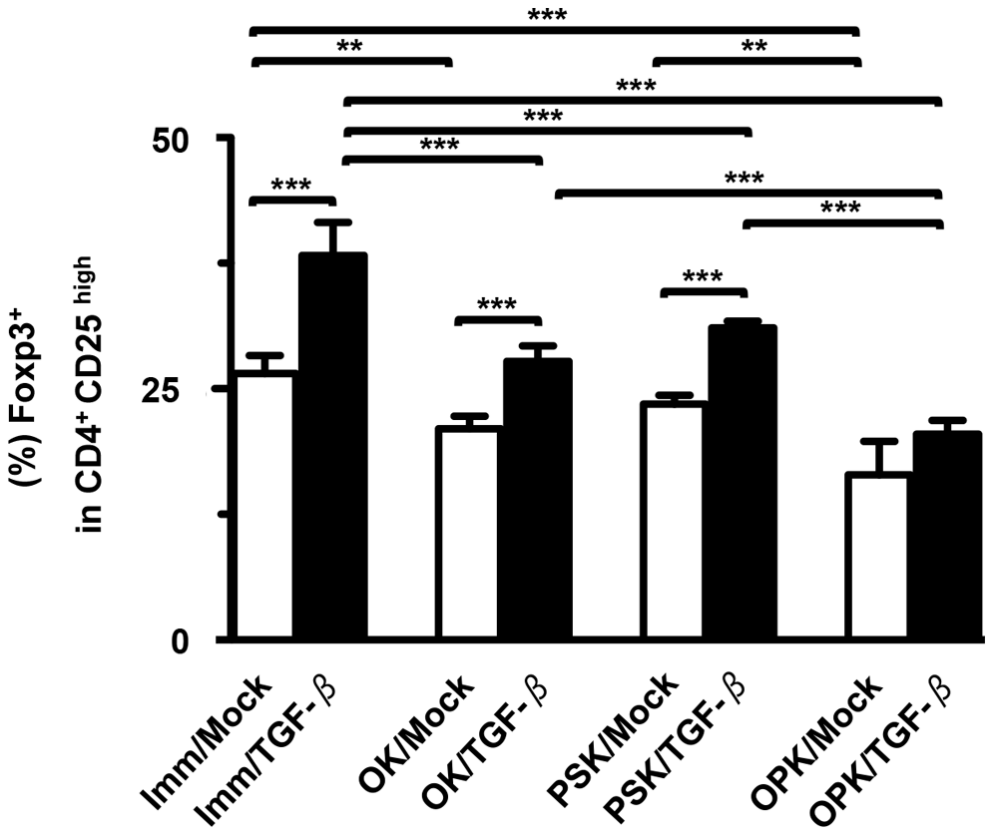
Generation of CD4^+^CD25^high^Foxp3^+^ T cells by DC/tumor. T cells (n = 5) were stimulated by eight types of DC/tumor (Imm/Mock, OK/Mock, PSK/Mock, OPK/Mock, Imm/TGF-β, OK/TGF-β, PSK/TGF-β, and OPK/TGF-β) and stained with Cy5-conjugated mAbs against CD4, FITC-conjugated mAbs against CD25, and PE-conjugated mAbs against Foxp3. To identify Foxp3^+^ cells in the CD4^+^CD25^high^ T cells, T cell populations were gated based on their forward- *vs.* side-scatter profile. CD4^+^CD25^high^ T cells were then analyzed for Foxp3 expression. The percentage of Foxp3^+^ cells among total CD4^+^CD25^high^ T cells is expressed as the mean ± SD. ***P<0.001; **P<0.01; *P<0.05.

Flow cytometry demonstrated that high expression of CD25 was observed in CD4^+^ T cells stimulated by OPK-DCs fused to all types of tumor cells (data not shown). This confirmed previous observations that the low affinity IL-2 receptor α-chain, CD25 was constitutively expressed on T regulatory cells (Tregs) and conventional TAA-specific CTLs [14]. Moreover, as shown in Figure 6, fusions generated with PANC/TGF-β and DCs increased the percentage of Foxp3^+^ cells in the CD4^+^CD25^high^ T cell population, compared with PANC/Mock. Importantly, combined OK-432- and PSK-activated DC/tumor showed superior efficacy in inhibiting CD4^+^CD25^+^Foxp3^+^ T cell generation, in comparison to those single activated or un-activated DC/tumor. In addition, there was no difference in the percentage of Foxp3^+^ cells in the CD4^+^CD25^high^ T cell population stimulated by DC/tumor generated with PANC-1 cells, compared to those generated with PANC/Mock (data not shown).

### MUC1-specific CTL responses by DC/tumor stimulated with combined OK-432 and PSK

**Figure 7:**
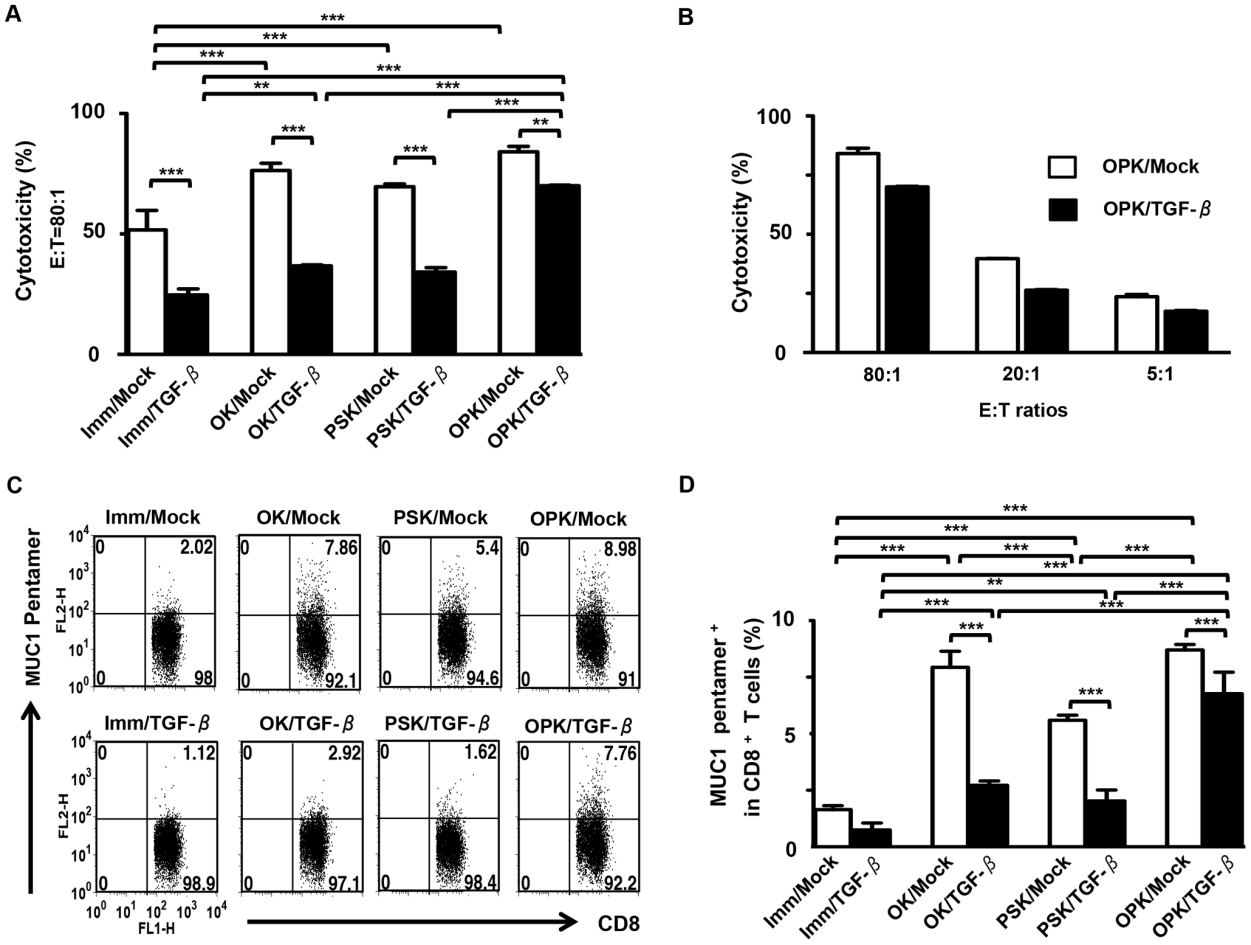
Induction of MUC1-specific CTL responses against PANC-1 cells by DC/tumor. (A) T cells (n = 3) were stimulated with the same number of fused cells that coexpressed both MUC1 and HLA-DR in eight types of DC/tumor in the same set of experiments. Stimulated T cells were incubated with PKH-26-labeled PANC-1 cells at a ratio of 80∶1 for cytotoxicity assays. (B) T cells (n = 3) stimulated by OPK/Mock or OPK/TGF-β were cocultured with PKH-26-labeled PANC-1 cells at the indicated E:T ratios. Percentage of cytotoxicity (mean ± SD) was determined by flow cytometry CTL assays. (C) T cells (HLA-A2^+^) (n = 5) stimulated by eight types of DC/tumor (HLA-A2^+^) (Imm/Mock, OK/Mock, PSK/Mock, OPK/Mock, Imm/TGF-β, OK/TGF-β, PSK/TGF-β, and OPK/TGF-β) were stained with FITC-conjugated mAbs against CD8 and PE-conjugated pentamer against HLA-A2/MUC1. The numbers of events in gated CD8^+^ T cells are shown. (D) The percentage of CD8^+^ T cells reactive to MUC1 among the whole CD8^+^ T cell population (n = 5) was shown as a percentage of the double-positive population (pentamer^+^CD8^+^) in the total CD8^+^ T cells. The results are expressed as the mean ± SD. ***P<0.001; **P<0.01; *P<0.05.

CTLs induced by any types of DC/tumor lysed PANC-1 cells (HLA-A2^+^MUC1^+^) (Fig. 7A) but not autologous monocytes (HLA-A2^+^MUC1^-^), MIA PaCa-2 (HLA-A2^-^MUC1^+^), and K562 (HLA-A2^-^MUC1^+^) cells (data not shown). The lysis against PANC-1 cells was inhibited by pre-incubation of PANC-1 cells with an anti-HLA-A2 mAb (data not shown). Although CTLs induced by Imm/Mock, OK/Mock, or PSK/Mock lysed PANC-1 cells, OPK/Mock was more effective at priming T cells to differentiate into CTLs (Fig. 7A and B). However, CTL activity against PANC-1 cells induced by OPK/TGF-β was significantly reduced compared to OPK/Mock (Fig. 7A and B), suggesting that the DC/tumor-derived active form of TGF-β1 reduced the efficacy of fusion-based cancer vaccines *in vitro*. Moreover, an increased percentage of MUC1-specific CD8^+^ T cells in the whole CD8^+^ T cell population was observed in the OPK/Mock, compared with OPK/TGF-β1 (Fig. 7C and D). In addition, there was no difference in CTL activity against PANC-1 cells induced by DC/tumor generated with PANC/Mock or PANC-1 cells (data not shown). CTLs specific for MUC1 were not detected in T cells stimulated by an unfused mixture of DCs and tumor cells (data not shown). Together, these findings indicate that HLA-A2 restrictive MUC1-specific CTLs that kill PANC-1 cells are efficiently induced by combined OK-432- and PSK-activated DC/tumor *in vitro*.

## Discussion

The data presented here show that combined TLR2/4-activated DC/tumor overcome immune-suppressive effect of TGF-β1 in comparison to those single activated or un-activated DC/tumor.

We attempted to prepare immunogenic DC/tumor that adhered to GMP processes. To ensure the delivery of a “cell drug” that is safe, reproducible, and efficient, FCS-independent tumor cells that could grow with human PPF were first established, as FCS has a possible risk of infection with pathogens of prion diseases for clinical use [11]. Moreover, both OK-432 and PSK that are GMP grade agents were used to stimulate DC/tumor. One of an important aspect of our work is its potential clinical relevance. Our findings that DCs activated with combined TLR2 and TLR4 are most active, indicate synergistic effects of dual-administered TLR agonists in the activation of DC functions. The efficient-activation of DCs by combined TLR2/4-agonists led us to speculate that DC/tumor generated in the presence of combined TLR2/4-agonists would be more effective than conventional un-activated fusions. Indeed, previous reports have demonstrated that activation of TLR4 signaling on DCs [15] and tumor cells [16] *in vitro*, even if transitory, induces the expression of IL-12p70 and pro-inflammatory mediators, respectively. In this study, OK-432-stimulated DC/tumor induced high levels of IL-12p70 production. OK-432 also promoted production of the active form of TGF-β1 from DC/tumor generated with PANC/TGF-β, however, the production was significantly inhibited when fusions were stimulated with combined OK-432 and PSK. Previously, it was shown that PSK can suppress production of IL-10, VEGF, and TGF-β1 [17]-[19], thus, DC/tumor activated with combined OK-432 and PSK may be stimulatory immunogenic due to decreased production of immune-suppressive molecules. Moreover, up-regulation of HSP90α in tumor cells and DCs by stimulation with combined OK-432 and PSK may provide proper costimulation during fusion process and can be expected to be involved in polarizing the T cell responses to a Th1-dominant state. However, high levels of the active form of TGF-β1 derived from DC/tumor inhibited the function of the fusions, even when stimulated with combined OK-432 and PSK. Therefore, TLR2/4-activation may be still insufficient to generate highly immunogenic DC/tumor when producing high levels of TGF-β1. In particular, TGF-β1 has a critical role in immune-suppressive mechanisms such as reducing the number and function of circulating DCs [20], generation of Tregs [21] and inactivation of CTLs [14], because of the suppressive effects of TGF-β1 on TLR2 and TLR4 signal transduction in DCs [22], [23]. Therefore, blockade of multiple immune-suppressive cytokines such as TGF-β1 from DC/tumor may be indispensable in adoptive immunotherapy.

In conclusion, our results demonstrate that combined TLR2/4-activated DC/tumor overcome immune-suppressive effect of TGF-β1 in comparison to those single activated or immature DC/tumor *in vitro*. However, tumor cells producing extremely high levels of TGF-β1 used for DC/tumor inhibit induction of efficient CTL responses. The alternative methods for inducing effective antigen-specific CTLs by highly immunogenic DC/tumor in the field of adoptive immunotherapy are currently under investigation.

## Supporting Information

Figure S1 **Establishment of a stable transfectant of PANC-1 cells expressing TGF-β1.** (A) TGF-β1 mRNA levels from a stable transfectant of PANC-1 cells expressing TGF-β1 was examined using real-time PCR in each clone. (B) Production of the active form of TGF-β1 in each clone was analyzed by ELISA.
(TIF)

Figure S2 **Characterization of a stable transfectant of PANC-1 cells expressing high levels of TGF-β1.** (A) The effects of total (latent and active form) of TGF-β1 from PANC/Mock or PANC/TGF-β#1 in the supernatants on Mv1Lu cells were analyzed. (B) The active form of TGF-β1 from PANC/Mock or PANC/TGF-β#1 produced in culture medium was measured by bioassay using Mv1Lu cells. The results are expressed as the mean ± SD.
(TIF)
