# Supplementary figures and images for: Combined TLR2/4-Activated Dendritic/Tumor Cell Fusions Induce Augmented Cytotoxic T Lymphocytes
Source: PLoS One. 2013 Mar 15;8(3):e59280. doi: 10.1371/journal.pone.0059280 (PMC3598755; doi:10.1371/journal.pone.0059280)

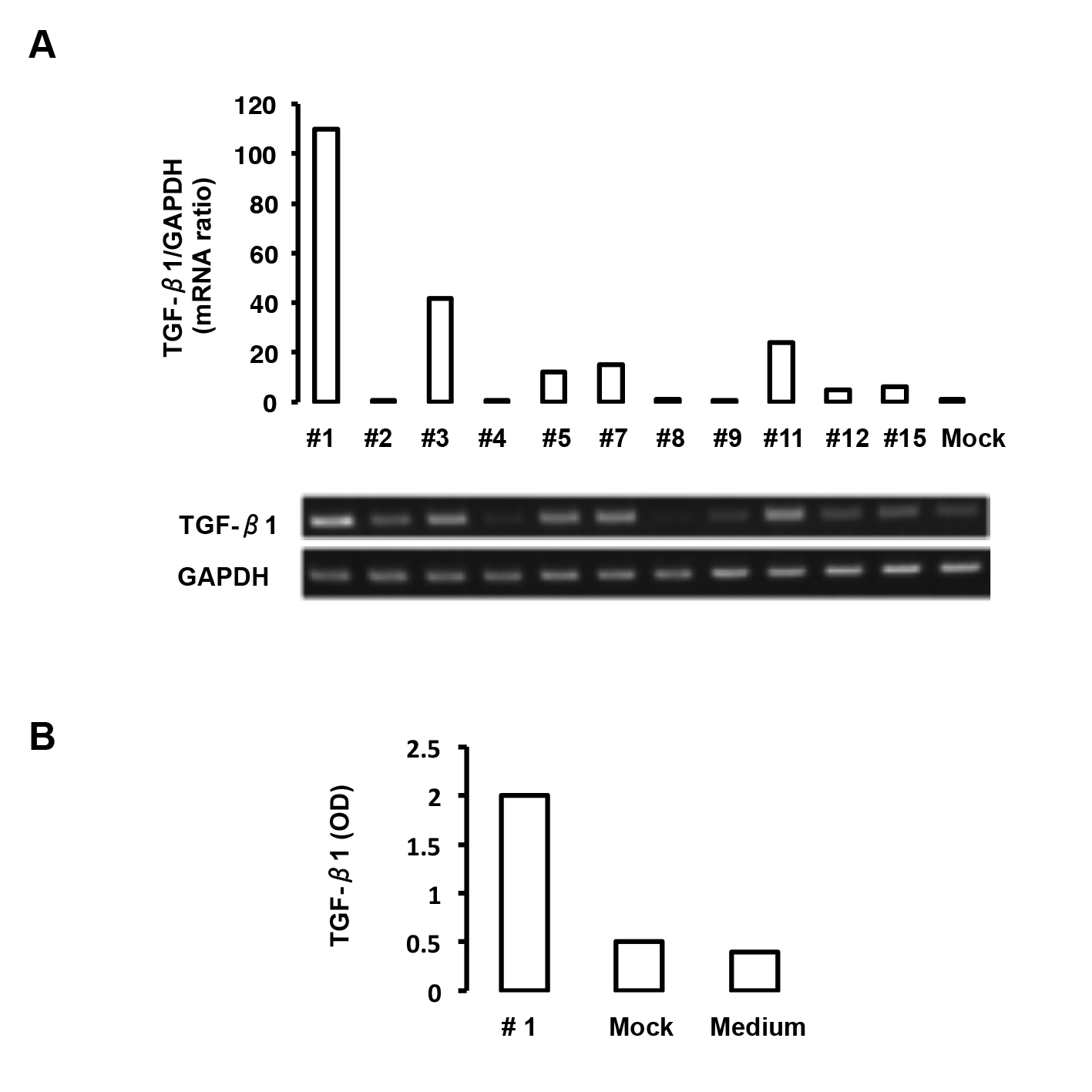

Supplement: Figure S1 — Establishment of a stable transfectant of PANC-1 cells expressing TGF-β1. (A) TGF-β1 mRNA levels from a stable transfectant of PANC-1 cells expressing TGF-β1 was examined using real-time PCR in each clone. (B) Production of the active form of TGF-β1 in each clone was analyzed by ELISA. (TIF) [file pone.0059280.s001.tif]

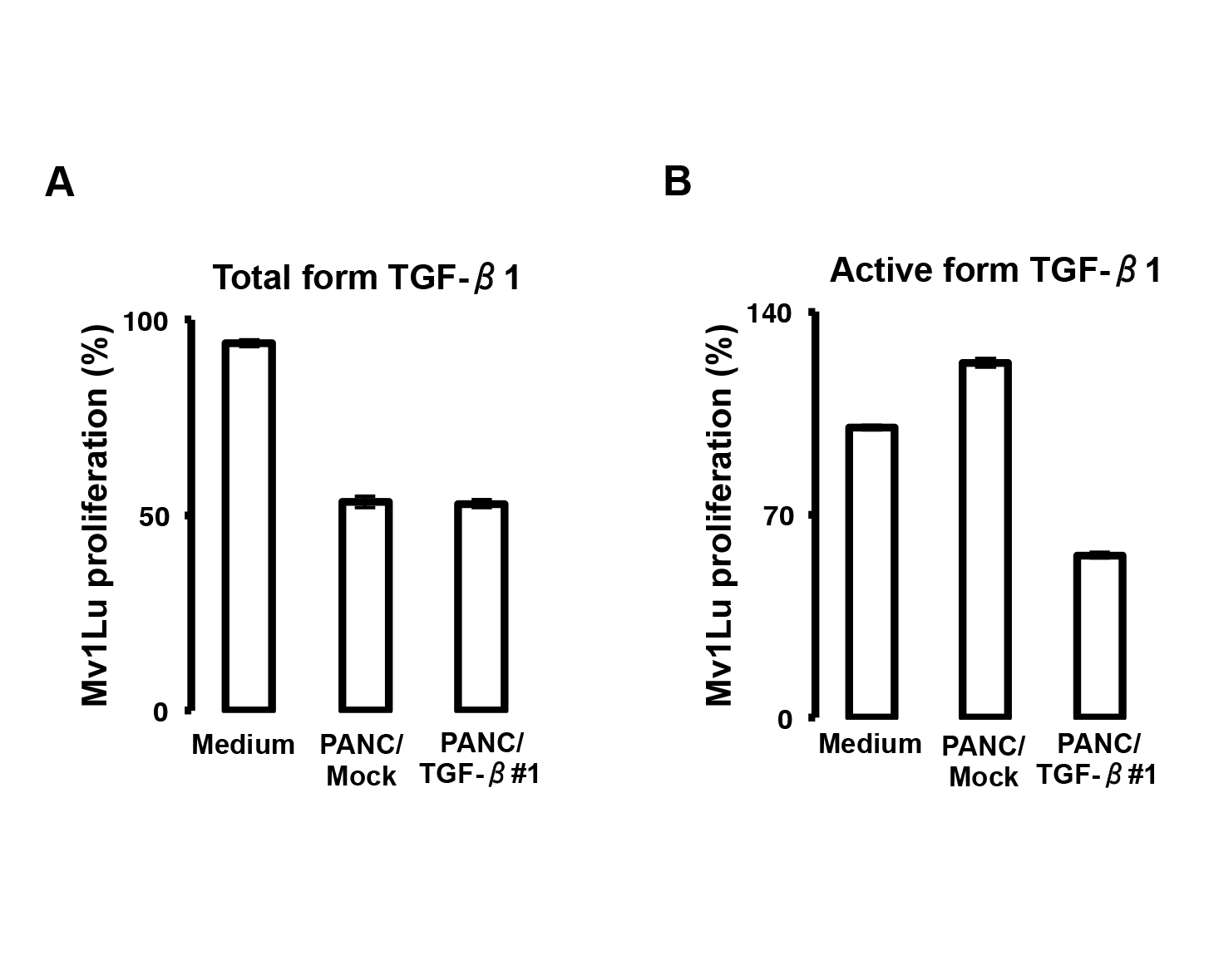

Supplement: Figure S2 — Characterization of a stable transfectant of PANC-1 cells expressing high levels of TGF-β1. (A) The effects of total (latent and active form) of TGF-β1 from PANC/Mock or PANC/TGF-β#1 in the supernatants on Mv1Lu cells were analyzed. (B) The active form of TGF-β1 from PANC/Mock or PANC/TGF-β#1 produced in culture medium was measured by bioassay using Mv1Lu cells. The results are expressed as the mean ± SD. (TIF) [file pone.0059280.s002.tif]
